# Fluid boluses and infusions in the early phase of resuscitation from septic shock and sepsis-induced hypotension: a retrospective report and outcome analysis from a tertiary hospital

**DOI:** 10.1186/s13613-024-01347-6

**Published:** 2024-08-15

**Authors:** Antonio Messina, Marco Albini, Nicolò Samuelli, Andrea Brunati, Elena Costantini, Giulia Lionetti, Marta Lubian, Massimiliano Greco, Guia Margherita Matronola, Fabio Piccirillo, Daniel De Backer, Jean Louis Teboul, Maurizio Cecconi

**Affiliations:** 1 IRCCS Humanitas Research Hospital, Via Manzoni 56, 20089 Rozzano, Milan Italy; 2 Department of Biomedical Sciences, Humanitas University, Via Levi Montalcini 4, Pieve Emanuele, Milan Italy; 3 Department of Intensive Care, CHIREC Hospitals, Université Libre de Bruxelles, Brussels, Belgium; 4 Paris-Saclay Medical School, Paris-Saclay University, Le Kremlin-Bicêtre, France

**Keywords:** Fluid therapy, Fluid bolus, Fluids, Electronic health records system, Fluid responsiveness, Hemodynamic monitoring

## Abstract

**Background:**

Fluid administration is the first line treatment in intensive care unit (ICU) patients with sepsis and septic shock. While fluid boluses administration can be titrated by predicting preload dependency, the amount of other forms of fluids may be more complex to be evaluated. We conducted a retrospective analysis in a tertiary hospital, to assess the ratio between fluids given as boluses and total administered fluid intake during early phases of ICU stay, and to evaluate the impact of fluid strategy on ICU mortality. Data related to fluid administration during the first four days of ICU stay were exported from an electronic health records system (ICCA®, Philips Healthcare). Demographic data, severity score, norepinephrine dose at ICU admission, overall fluid balance and the percentage of different fluid components of the overall volume administered were included in a multivariable logistic regression model, evaluating the association with ICU survival.

**Results:**

We analyzed 220 patients admitted with septic shock and sepsis-induced hypotension from 1st July 2021 to 31st December 2023. Fluid boluses and maintenance represented 49.3% ± 22.8 of the overall fluid intake, being balanced solution the most represented (40.4% ± 22.0). The fluid volume for drug infusion represented 34.0% ± 2.9 of the total fluid intake, while oral or via nasogastric tube fluid intake represented 18.0% ± 15.7 of the total fluid intake. Fluid volume given as boluses represented 8.6% of the total fluid intake over the four days, with a reduction from 25.1% ± 24.0 on Day 1 to 4.8% ± 8.7 on Day 4. A positive fluid balance [OR 1.167 (1.029–1.341); p = 0.021] was the most important factor associated with ICU mortality. Non-survivors (n = 66; 30%) received a higher amount of overall inputs than survivors only on Day 1 [2493 mL vs. 1855 mL; p = 0.022].

**Conclusions:**

This retrospective analysis of fluids given over the early phases of septic shock and sepsis-induced hypotension showed that the overall volume given by boluses ranges from about 25% on Day 1 to about 5% on Day 4 from ICU admission. Our data confirms that a positive fluid balance over the first 4 days of ICU is associated with mortality.

**Supplementary Information:**

The online version contains supplementary material available at 10.1186/s13613-024-01347-6.

## Introduction

Fluid administration is the first line treatment of critically ill patients admitted to the intensive care unit (ICU) with septic shock, aiming to increase venous return, stroke volume and, consequently, cardiac output and tissue oxygen delivery [1–5]. However, the modality and the overall volume of fluids administration during sepsis-induced organ dysfunction is still debated and characterized by a significant heterogeneity in practices [6, 7]. In fact, the initial strong recommendation of 2016 Surviving Sepsis Campaign (SSC) guidelines of giving at least 30 mL/kg of crystalloids for initial resuscitation of patients with sepsis-induced hypoperfusion [8] has been downgraded to a weak recommendation in the 2021 version [9]. Recent randomized-controlledtrials in patients with sepsis-induced hypotension (averaged mortality of 14%) [10] and in patients with septic shock (average mortality of 42%) [11], showed that a “restrictive” fluid policy was non-inferior to a “liberal” one. However, patients were randomized into the two groups not necessarily in the early resuscitation phase, and after receiving up to 2 L [10] or nearly 3 L [11] of initial fluid resuscitation [10, 11]. The same lack of potential benefits of one regimen over the other has been found in a recent meta-analysis of 13 RCTs including almost 4000 patients [12]. Moreover, the modality of fluid administration is highly variable among ICUs. As a confirmation, a recent prospective multicenter cohort study conducted in 30 ICUs in France and Spain found that a center effect was the strongest factor associated with the volume of fluids administered over 24 h following admission [13].

Several methods are currently used to predict fluid responsiveness and, accordingly, to administer boluses of fluids to improve tissue perfusion [7, 14, 15]. However, less attention is usually paid to the other components of fluid intake, usually predominant ones [16], including fluid infusions given as maintenance, creep fluids (fluids given with medication and flushes) and nutrition (enteral and parenteral) [16, 17]. Of note, in a single center ICU trial, maintenance and replacement fluids accounted for 24.7% of the mean daily total fluid volume, and fluid creep represented the 32.6% of the mean daily total fluid volume [16].

Overall, the two main issues about ICU fluid administration regard the appropriate overall fluid infusion during the whole stay and to the modality of using fluids according to the pathophysiological changes in each patient during and after resuscitation, aiming at maintaining adequate organ perfusion during initial phases of hemodynamic instability and limiting or removing inappropriate fluid intake later on [i.e*.* a conceptual model indicated by the acronym R.O.S.E. (Resuscitation, Optimization, Stabilization, Evacuation)] [18].

Since 2021, all the 14 beds of the ICU of the Humanitas Research Hospital (Rozzano, Milan), a tertiary hospital in the North of Italy, have been equipped with an ICU clinical information system (ICIS) developed by Philips®, called Intellispace Critical Care and Anesthesia (ICCA®). ICCA® allows a precise prescription of all the medical treatments, including fluids (administration route, doses, administration times, and duration), as well as the automatic collection of patient’s vital parameters from bedside monitors and ventilators.

We conducted a retrospective analysis of the use of fluids in our ICU, with the primary aim of mapping out the detailed proportion between fluid boluses and other types of fluid infusions in the early phases (i.e. ICU days 1–4) of patients admitted for septic shock and sepsis-induced hypotension. We secondarily described the different types of fluids given (i.e. albumin, normal saline, Ringer, balanced solution, others) and the four main drug categories (i.e. vasoactive, sedative/analgesic, anti-infective and other drugs). Finally, we assessed whether fluid administration modality and fluid types may impact ICU mortality.

## Materials and methods

The retrospective use and analysis of data has been approved by the local ethical committee of Humanitas Research Hospital (acknowledgment for the study n 26/23). The data were collected in a structured and representative manner according to the Declaration of Helsinki. This study is reported according to the “Strengthening the Reporting of Observational Studies in Epidemiology (STROBE)” statement guidelines for observational cohort studies (Supplementary File 2 in the Supplementary Materials) [19].

### Aims of the study

The primary aim of this study was to address the detailed proportion between fluid boluses and infusions in the early phase of ICU patients admitted for septic shock. Secondary aims were (1) to describe the different types of fluids given and the main drug categories; (2) to assess whether fluid administration modalities and fluid types may affect ICU mortality.

### Patient selection

ICU patients have been selected from the PROSAFE (case report form) eCRF, which is a multilingual evolution of the Margherita Project eCRF, adopting a modular structure designed to collect basic data (“core” module) into specific modules called “petals” [20]. Each patient admitted to the ICU from the emergency department, medical wards, or operating rooms of Humanitas Research Hospital was recorded into the PROSAFE eCRF and then stratified according to the appropriate “petal”. We then selected patients admitted with the diagnosis of “sepsis” and “septic shock”, and receiving continuous norepinephrine within the first 6 h of ICU admission. Finally, we exported demographic data, baseline characteristics, severity scores, type and source of infection on a dedicated Excel® (Excel 2011; Microsoft, Redmond, WA) spreadsheet.

All the other data were extracted from ICIS ICCA® (Philips Healthcare, Amsterdam, Holland) of the ICU of Humanitas Research Hospital from 1st July 2021 (data of full integration between ICCA® system and ICU infusion pumps) to 31st December 2023.

We excluded from the analysis all the patients who died or were discharged early after ICU admission (i.e., within 24 h), and those with missing data regarding norepinephrine or fluid infusion (implying a potential error in the demographic ICU admission data recording, or duplicated patients with no data recorded). Patients have then been classified in two groups: (1) septic shock, if at ICU admission they received norepinephrine infusion (any dose) and had lactate level ≥ 2 mmol/L, as recorded by the first arterial blood gas analysis present on ICCA®; (2) sepsis-induced hypotension, if at ICU admission, they received norepinephrine infusion (any dose) but had lactate level < 2 mmol/L, as recorded by the first arterial blood gas analysis present on ICCA®.

### Fluid data extraction

Overall fluid data assessment has been performed considering specific ICCA® data outputs (in mL) during the first four days from ICU admission. ICCA® automatically records all the prescriptions, including fluids, being also integrated with infusion pumps. For this study, we extracted the following items:

- Fluid balance: the overall difference between *inputs* (any type, including maintenance fluids) and *outputs* [any type, including urine output and other outputs (i.e. drainages, enteral feeding regurgitation etc.)]. Losses through perspiration were not estimated.
- Fluids given each day:
  - Fluid boluses: Intravenous (IV) fluid infused as aliquot. The search was restricted to prescriptions of 250 mL, 500 mL, 1000 mL of fluid boluses, which are the standard prescriptions for boluses adopted in our ICU.
  - Fluid infusions: ANY type of fluid given not as a bolus or drugs (see above and below).
  - Drugs: we extracted 223 infusive drugs from the overall list available for prescription on ICCA® (see Supplemental Table 1 in the Supplementary File 1), and merged them into four main subgroups of IV drugs, either given by infusive pumps or not:
    - IV Vasoactive drugs
    - IV Sedative/analgesic
    - IV Antinfective drugs
    - IV Other drugs
- Enteral feeding [by nasogastric tube (NGT)], or *per os* intake.

Moreover, the type of fluids used as boluses or continuous infusions or diluent of other drugs has been then stratified according to four main subgroups:

- Albumin
- Normal Saline
- Balanced solution (Reidrante III, Baxter SpA; Ringer lactate or acetate)
- Other fluids (i.e. any type of glucosate, bicarbonate; blood products, parenteral nutrition)

### Statistical analysis

Information has been retrieved from the MS SQLServer Data Warehouse (DWH) of ICCA® and extracted using Python® (Python Software Foundation). Since ICCA® automatically calculates the exact 24 h fluid balance at 06.00 a.m. of every day, the first day of ICU stay corresponded to the time spent in ICU before the first fluid balance. Before building the final dataset, we checked data quality and consistency by calculating the difference between the overall intake of IV inputs extracted by the system and the reference label “overall daily intake” at 06.00 a.m. of each day, since this value is daily calculated by ICCA® by automatically summing all the IV mL administered before that timepoint. A difference of ± 5% was considered acceptable; patients showing a bigger difference were manually checked and data were eventually corrected. At end of the data quality check, the median (IQR) percentage difference between the “overall daily intake” and extracted data at end of data quality check was 0% [− 0.9 to 1.0] and the final dataset was structured.

For the purpose of evaluating the volume of fluids infused before ICU admission, we checked the prescriptions recorded in the software adopted in our emergency department (PIESSE®—Dedalus, Italy), medical wards and operating rooms (WHOSPITAL ®—Dedalus, Italy).

Normal distribution of continuous variables was evaluated employing the d’Agostino-Pearson test and results are reported in the whole manuscript as median (25th–75th interquartile range) or mean [standard deviation (SD)], as appropriate. Continuous variables were compared using Mann–Whitney U test or Student-t test, while dichotomous or categorical variables were compared utilizing the chi-square test for comparison of proportions, as appropriate.

Finally, age, body mass index, Sequential Organ Failure Assessment (SOFA) score and norepinephrine dose at ICU admission, overall fluid balance and the overall volume of each type of fluid given, expressed as the ratio between each component with respect to the overall fluid intake over the four considered days, were included in a multivariable logistic regression model, evaluating the association with ICU mortality. Variables were assessed for collinearity prior to inclusion in the model and only independent variables were included. The model was constructed only considering patients who were still present in ICU at day 4, to avoid confounding factors on fluid balance due to different ICU stays.

Data analysis was conducted using Python® and GraphPad PRISM V8 (GraphPad Software Inc., San Diego, CA, USA). A p-value < 0.05 was considered statistically significant for all the comparisons.

## Results

**Fig. 1:**
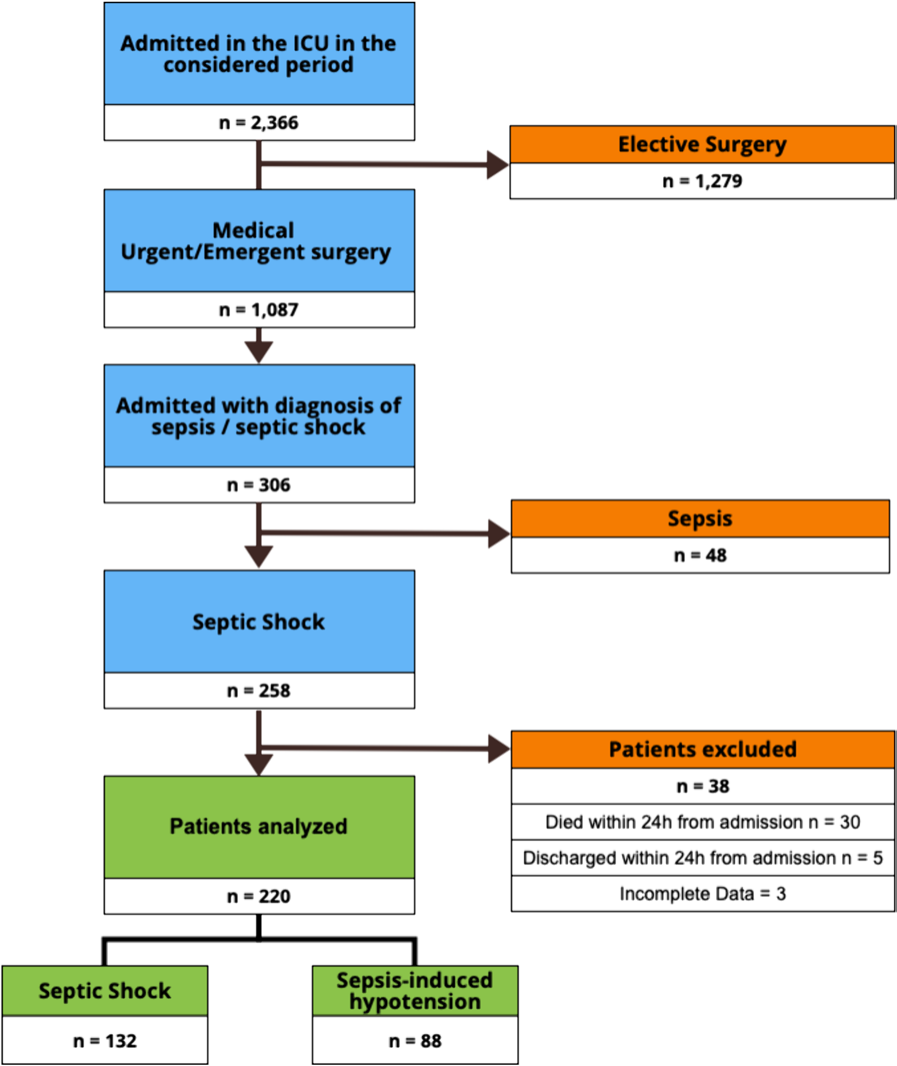
Flow of the patients. Incomplete data refer to those patients in whom data regarding norepinephrine or fluid infusion were missing

In the recording period, 258 patients (23.7% of those not admitted for elective surgery) were admitted with diagnosis of septic shock (n = 132; 60.0%) and sepsis-induced hypotension (n = 88; 40.0%) (Fig. 1). After excluding 38 patients for early (< 24 h) death (n = 30) or discharge (n = 5) and for incomplete or corrupted data input (n = 3), 220 patients have been finally analyzed, with 66 patients (30.0%) who died. The initial number of generated ICCA® raw cells after the first query associated to these patients’ ICU stay was 108,231, while the number extracted and analyzed in the final dataset after subdividing and merging the different types of inputs and outputs was 31,900.

Respiratory infections were the most common reason for ICU admission (30.9%) (Table 1), followed by gastrointestinal infections (30.0%). Survivors had a mean ICU stay of 11.5 ± 1 days and hospital stay of 23.5 ± 24.5 days.

**Table 1:** Patients’ characteristics at enrolment

General characteristics	Overall (n = 220)	ICU survivors (n = 154)	ICU non-survivors (n = 66)	p value*
Age (year)	68 [57–75]	67 [57–74]	71 [60–77]	0.06
Gender (M/F)	142/79	104/50	37/29	0.12
Body mass index (Kg/m^2^)	25.7 [23.0–29.4]	25.7 [23.4–29.7]	25.2 [22.2–29.0]	0.24
SAPS II score	45 [35–56]	42 [31–50]	56 [44–67]	< 0.0001
SOFA score	8 [6–11]	8 [6–10]	10 [7–13]	< 0.0001
Type of admission (n; %)				
Medical	134; 60.9	91; 59.1	43; 65.1	0.45
Urgent/emergent surgery	86; 39.1	63; 40.9	23; 34.9	
Source of infection				
Confirmed (n; %)				
1. Respiratory Infection	68; 30.9	44; 28.6	21; 31.8	0.63
2. GI infection	66; 30.0	46; 29.9	20; 30.3	0.99
3. Urinary tract	20; 9.1	17; 11.0	3; 4.5	0.20
4. Primary bacteremia	16; 7.3	11; 7.1	5; 7.6	0.99
5. Endocarditis	8; 3.6	5; 3.3	3; 4.5	0.69
6. Meningitis	3; 1.4	0; 0.0	3; 4.5	0.02
Suspected or unknow (n,%)	39; 17.7	31; 20.1	11; 16.8	0.71
Lactate at ICU admission (mmol/L)	2.5 [1.5–4.2]	2.4 [1.5–3.7]	2.7 [1.7–5.2]	0.06
NE at ICU admission (mcg/kg/min)	0.4 [0.2–0.9]	0.4 [1.5–0.8]	0.6 [0.27–1.6]	0.0007

Data presented as median (25th–75th IQR). *SAPS* simplified acute physiology score, *SOFA* sequential organ failure assessment, *NE* norepinephrine, *ICU* intensive care unit, *GI* gastrointestinal tract. Respiratory infections included: pneumonia (any type including COVID; upper and lower airways infection; pleuritis and pleural empyema). GI infections included gastroenteritis, peritonitis (any type), cholangitis, and cholecystitis

^*^p values refer to the comparison between survivors and non-survivors

The time spent in ICU before the first fluid balance assessment (considered as Day 1 of ICU stay, see Methods) corresponded to a median (IQR) of 13 h (10–17) hours, and was comparable between survivors and non-survivors [13 h (9–16) vs. 14 h (10–18); p = 0.15)].

### Fluid boluses vs. overall fluid infusions

**Fig. 2:**
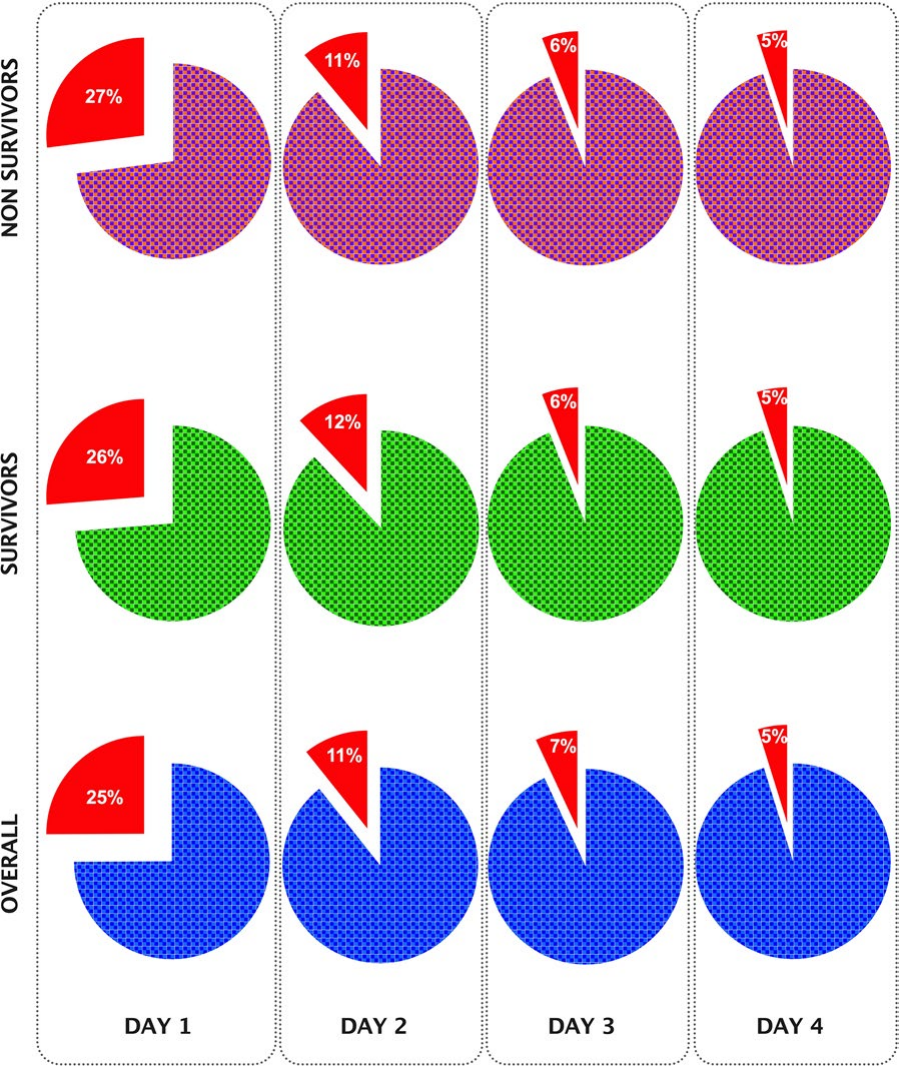
Pie charts regarding the percentage of fluids given as bolus as compared to the overall daily intake over the first 4 days from ICU admission, in survivors, non-survivors and overall population

Considering all the patients, fluids given as boluses represented 25.1% ± 24.0 of the overall fluid infusions on Day 1, 10.7% ± 13.1 on Day 2, 6.7% ± 10.6 on Day 3, and 4.8% ± 8.7 on Day 4 (Fig. 2). The percentage of fluids given as boluses on Day 1 was significantly higher (p < 0.001) as compared to all the other considered days. Overall, 8.6% ± 10.5 of fluids given over the first four days were administered as boluses (Supplemental Fig. 1 in the Supplementary File 1). The mean fluid volume given as boluses was 594 mL ± 680 on Day 1, 386 mL ± 511 on Day 2, 187 mL ± 325 on Day 3, and 135 mL ± 255 on Day 4. In non-survivors, the overall mean fluid volume given as boluses over the first 4 days was larger as compared to survivors [1436 mL ± 923 vs. 1117 mL ± 1049; p = 0.005], being larger on days 1–2 and 4 (Supplemental Fig. 1 in Supplementary File 1).

### Volumes and types of fluid infused on Days 1–4

It has been possible to retrieve the exact volume of fluids received before ICU admission of 162 (73.6%) of the included patients. The median (IQR) fluid volume given was 1500 (800–2463) mL, corresponding to a median (IQR) of 19.8 (10.1–29.6) mL/kg, and was comparable between survivors and non-survivors [1500 mL (800–2113) vs. 1117 mL (787–2500); p = 0.59].

As shown in Table 2, the daily fluid balance was higher on Day 1 and Day 3 in non-survivors as compared to survivors. Median fluid balance at Day 4 was neutral for both subgroups. Non-survivors received a higher volume of overall inputs as compared to survivors only on Day 1 [2493 (1489–2963) mL vs. 1855 (1167–2638) mL; p = 0.022].

**Table 2:** Daily fluid balance, outputs and inputs

	DAY 1	DAY 2	DAY 3	DAY 4	Overall
Fluid balance (mL)					
Survivors	860 [250–1651]	973 [174–2309]	171 [− 337–827]	0 [− 543–727]	2340 [214–4162]
Non-survivors	1655 [679–2462]	799 [0–2509]	520 [0–1580]	0 [0–968]	4012 [1563–6495]
Overall	992 [330–1945]	909 [34–2368]	265 [− 259–1000]	0 [− 476–800]	2543 [869–4801]
p-value*	< 0.001	0.91	0.007	0.06	0.001
Overall input (mL)					
Survivors	1855 [1167–2638]	3003 [2191–3897]	2338 [1675–2838]	2254 [1497–2806]	9276 [7469–11747]
Non-survivors	2493 [1489–2963]	2698 [1851–3649]	2525 [1434–3015]	2146 [0–3058]	9507 [6527–11517]
Overall	2006 [1191–2783]	2907 [2152–3783]	2388 [1650–2883]	2242 [1384–2888]	9307 [7453–11552]
p-value*	0.022	0.08	0.68	0.68	0.73
Overall output (mL)					
Survivors	830 [453–1631]	1840 [1139–2609]	2010 [1355–2710]	2110 [1486–2898]	7140 [5446–9024]
Non-survivors	720 [347–1263]	1450 [810–1989]	1457 [581–2333]	1805 [0–2323]	6140 [3053–7586]
Overall	790 [392–1368]	1715 [1060–2490]	1890 [1183–2578]	1985 [1233–2718]	6885 [4726–8780]
p-value*	0.08	0.001	0.001	0.003	< 0.001

Data presented as median (25th-75th IQR); *p values refer to the comparison between survivors and non-survivors

**Fig. 3:**
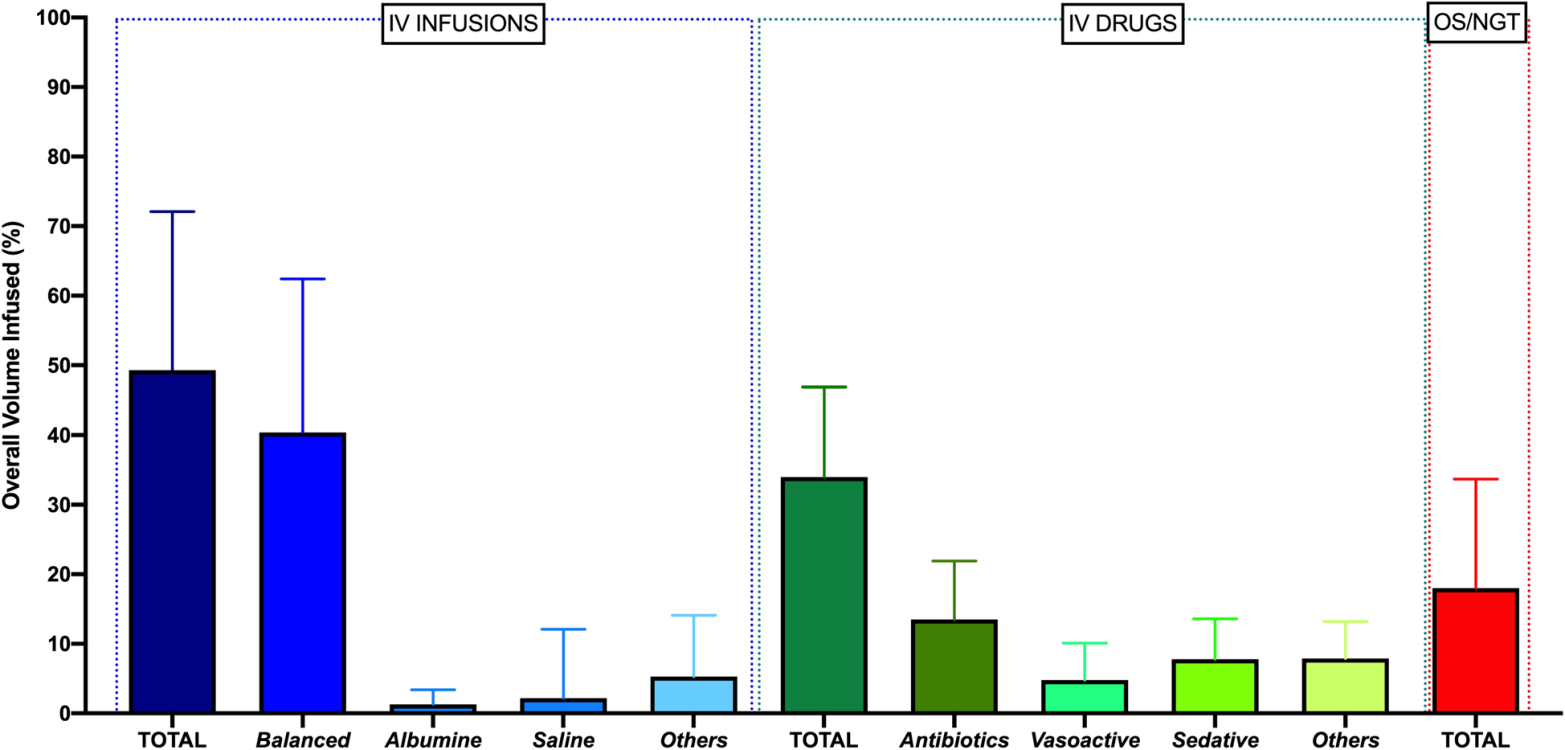
Different types of fluids and drugs given during the ICU stay, expressed as percentage with respect to the overall volume infused. *NGT* nasogastric, *IV* intravenous, *OS* per os

As shown in Fig. 3 and Supplemental Tables 2 and 3 in Supplementary File 1, IV infusions (including boluses) represented 49.3% ± 22.8% of the overall fluid intake, being balanced solution the most represented (40.4% ± 22.0%). The fluid volume associated with IV drugs administration represented 34.0% ± 2.9% of the total fluid intake, being balanced solution the most represented (13.5% ± 8.4%), while *per os*/NGT intake represented 18.0% ± 15.7% of the total.

### Variables associated to ICU mortality

The model was constructed on the data of 160 patients still present in ICU at Day 4. As shown in Table 3, a positive fluid balance [OR 1.167 (1.029–1.341); p = 0.021] was the strongest factor associated with ICU mortality. Norepinephrine dose at ICU admission (p = 0.048) and a higher percentage of used saline (p = 0.042) were also associated with ICU mortality, with borderline significance.

**Table 3:** Multivariable logistic regression model

Variable	Coefficient	Std Error	Odds Ratio	p-value
Fluid balance (tot)	0.154	0.67	1.167 (1.029–1.341)	**0.021**
SOFA score at ICU admission	0.085	0.07	1.089 (0.951–1.254)	0.221
Age	0.027	0.018	1.027 (0.993–1.067)	0.136
BMI	− 0.074	0.044	0.929 (0.846–1.008)	0.094
Norepinephrine dose at ICU admission	0.016	0.008	1.016 (1–1.033)	**0.048**
Boluses (%)	− 0.232	0.167	0.793 (0.568–1.106)	0.166
Balanced Solution (%)	− 0.08	0.089	0.923 (0.772–1.098)	0.369
Normal Saline (%)	0.259	0.127	1.295 (1.009–1.672)	**0.042**
Albumin (%)	0.173	0.153	1.189 (0.882–1.617)	0.259

In the regression model, the values reported as percentage refer to the ratio between the volume of the specific fluid administered over DAYS 1–4, with respect to the overall fluid infusion

Statistically significant values are reported in bold

*SOFA score* Sequential Organ Failure Assessment score, *BMI* body mass index, *ICU* intensive care unit

## Discussion

In this study, we reported the different qualitative and quantitative components of fluid administration over the first 4 ICU days of patients admitted to a tertiary hospital with septic shock. To the best of our knowledge, this is the first study accurately reporting the detailed proportion between fluid boluses and infusions in this context.

The main results can be summarized as follows: (1) the overall amount of fluid given as boluses was the vast minority of total fluid intake; overall, less than 10% of fluids given over the first four days of ICU stay have been administered as boluses; (2) the overall fluid balance was about 1 L positive over the first 2 days from ICU admission and tended to be neutral on Day 3 and Day 4; non-survivors received a higher intake only on Day 1, as compared to survivors; (3) in non-survivors, the volume of fluids given as boluses over the first 4 days was larger as compared to survivors; (4) a positive fluid balance over the first four days of ICU stay was the variable most associated with mortality.

The discussion regarding fluid administration in ICU patients with septic shock is still a hot topic. An abundant literature over the last two decades investigated different aspects such as effective volume, flow and pressure response, and infusions modalities [6, 14, 15, 21, 22]. However, data regarding the use of fluid boluses in early phases of resuscitation are scarce. Van Regenmortel et al. reported the volume, sodium, and chloride burdens imposed by every fluid source administered to a large mixed population of 14,654 critically ill patients, with an overall 28-days ICU mortality of 11% [16]. The authors reported that “resuscitation fluids” represented only 6.5% of overall fluid administrations in non-selected critically ill patients. However, when focusing on medical patients and on those admitted after emergency surgery, fluid boluses represented 21.1% of the overall fluid administrations [16]. This is consistent with our data, showing that in septic shock patients fluid boluses represent 25% of overall infusions on Day 1, progressively decreasing to lower values over the following days.

Our data show that fluid boluses represent the minority of fluid administrations. This finding should be coupled to fluid responsiveness assessment in real life at the bedside. In fact, since the hemodynamic response to a fluid bolus in septic shock is known to fade rapidly [23], all the boluses should be infused only if fluid responsiveness has previously been assessed. Again, data about the systematic assessment of fluid responsiveness are lacking, despite a recent secondary analysis of the ANDROMEDA-SHOCK trial reporting that systematic assessment allowed determination of fluid responsiveness status in more than 80% of patients with early septic shock [23]. So, the small amount of volume given by fluid boluses could be related to the presence of fluid unresponsiveness, assessed at the bedside.

Interestingly, after Day 1, the overall volume of resuscitation fluids given as boluses (on average between 200 and 300 mL) was overall comparable to all the other medications infused by pumps or not (including antibiotics and sedative drugs) (see also Supplementary Materials).

Since the volume of fluids given by fluid boluses was progressively reduced, and the overall positive fluid balance was an independent factor associated with ICU mortality, our results emphasize the need of quantifying and controlling all the other forms of therapeutic infusions, including diluted drugs and pumps’ infusions, to minimize the amount of unnoticed and potentially harmful fluid intake. These results encourage conducting further studies to evaluate whether the modality of fluid administration, and the overall input volume in the different R.O.S.E. model part may impact clinical ICU outcomes.

In ICU patients, a fluid bolus is usually performed by infusing 500 mL of fluid [24], most often a crystalloid solution, in less than 20 min. This is also the median volume adopted in daily clinical practice [25], whereas smaller fluid boluses of about 250 mL are usually adopted in high-risk surgical patients undergoing goal-directed therapy optimization [26]. The policy of our ICU is to give fluids by pumps, with the purpose of keeping a constant rate and ensure a precise fluid balance, since all the pumps are integrated in the ICCA® system. However, in the most severe patients, at ICU admission, fluids are sometimes given by bags or even manually, to ensure a faster rate. Nevertheless, these aliquots are considered in the daily fluid balance as boluses too, since infused in the fixed aliquots of 250, 500, or 1000 mL. The rate and the overall fluid volume given as boluses are known to affect cardiac output responses [27, 28], but probably not clinical outcomes [29].

The value of the regression model is limited by the relatively small number of patients included and the functional overlap of different variables (i.e. the percentage of fluid and the overall fluid balance). Moreover, we considered only the first four days from ICU admission, considering the mean plasmatic level of lactate of the included population, which was normalized at Day 4.

Fluid overload is independently associated to worse outcomes of ICU patients [30–32] and, intuitively, the most severe patients would also receive more fluids in the resuscitation phase. However, our data confirm that the fluid strategy should be carefully titrated in patients with septic shock. Moreover, we found a borderline significance related to an increased risk of ICU mortality in those patients receiving a higher percentage of saline with respect to the overall amount of fluids administered. Recent findings suggest that the probability that using balanced solutions vs. saline in the ICU reduces in-hospital mortality is high [33], and the European Society of Intensive Care Medicine clinical practice guideline on fluid therapy in adult critically ill patients suggests balanced solutions over saline in both general and septic ICU population [34].

### Limitations

The main limitation of this study is that the retrospective design of the analysis limits the generalizability of our results, which are also affected by the local resources of our hospital, as compared to others. This is specifically true for the use of an ICIS allowing the precise data recording of all the vital parameters and medications during the whole ICU stay. For this reason, outcomes and possible associations should be considered with caution.

Data obtained by from ICCA® are accurate since specific exact queries are adopted for data extraction. This is one strength of this study. However, this may introduce potential biases. Moreover, the classification of “Day 1 of ICU admission” has been necessarily defined according to the first fluid balance extracted by ICCA® at 06.00 a.m., and not as the first 24 h from ICU admission. For this reason, the hours spent in ICU during Day 1 by the patients may differ and, accordingly, the volume of fluids received may differ as well. However, the number of hours considered as the Day 1 was comparable between survivors and non-survivors, minimizing the risk of this bias in data analysis.

The classification of fluid bolus as an infusion of three fixed quantities of fluids (250 mL, 500 mL or 1000 mL) corresponds to our local practice and allowed a precise definition of the quantity given by using ICCA®. However, it is still possible that a minority of fluid boluses of different volumes (i.e. 300 mL) could be miscalculated as infusions, not matching the initial classification. It is considered good clinical practice in our center to assess fluid responsiveness before fluid boluses administration, especially after Day 1 from ICU stay. However, the number and the types of assessments are not automatically extractable. Moreover, the reason for administering fluids is not recorded on ICCA, so this information cannot be provided.

Also, the classification of “other fluids” is arbitrary. However, the overall volume of this fluid subgroup is very small and further sub-classifications would not add valuable information.

Finally, we did not have access to the daily weight of the patients, so that the fluid balance was calculated by considering inputs and outputs, and some imprecisions in the fluid balance records (e.g. regurgitation, drainages) are likely to have occurred.

## Conclusions

This retrospective analysis based on a precise evaluation of fluids given over the early phases of septic shock and sepsis-induced hypotension in an Italian tertiary hospital showed that the overall volume of fluids given as boluses is small compared to the total fluid intake, ranging from about 25% on Day 1 to about 5% on Day 4 from ICU admission. Moreover, our data confirm that a positive fluid balance over the first 4 days of ICU is associated to mortality, and suggest a potential harmful signal regarding saline infusion.

### Supplementary Information

Supplementary Material 1.

Supplementary Material 2.

## Data Availability

The datasets used and/or analyzed during the current study are available from the corresponding author on reasonable request.

## Abbreviations

ICU: Intensive care unit
ICIS: ICU clinical information system
CRF: Case report form
NGT: Nasogastric tube
