# Supplementary material for: Fluid boluses and infusions in the early phase of resuscitation from septic shock and sepsis-induced hypotension: a retrospective report and outcome analysis from a tertiary hospital
Source: Ann Intensive Care. 2024 Aug 15;14:123. doi: 10.1186/s13613-024-01347-6 (PMC11327232; doi:10.1186/s13613-024-01347-6)
Supplement: Supplementary file 1 — Supplementary Material 1. [file 13613_2024_1347_MOESM1_ESM.docx]

**Fluid boluses and infusions in the early phase of resuscitation from septic shock: a retrospective report and outcome analysis from a tertiary hospital.**

Antonio Messina^1,2^; Marco Albini^1^(^); Nicolò Samuelli^1^; Andrea Brunati^2^; Elena Costantini^1^, Giulia Lionetti^1,2^; Marta Lubian^1,2^; Massimiliano Greco^1,2^, Guia Margherita Matronola^2^; Fabio Piccirillo^1^; Daniel De Backer^3^, Jean Louis Teboul^4^, Maurizio Cecconi^1,2^.

^1^IRCCS Humanitas Research Hospital, via Manzoni 56, 20089 Rozzano - Milan, Italy.

^2^Department of Biomedical Sciences, Humanitas University, via Levi Montalcini 4, Pieve Emanuele - Milan, Italy.

^3^ Department of Intensive Care, CHIREC Hospitals, Université Libre de Bruxelles, Brussels, Belgium

^4^Paris-Saclay Medical School, Paris-Saclay University, Le Kremlin-Bicêtre, France

**Corresponding author:**

Antonio Messina; Department of Anesthesia and Intensive Care Medicine

IRCCS Humanitas Research Hospital, via Manzoni 56, 20089 Rozzano - Milan, Italy;

Email: antonio.messina@humanitas.it

Tel: +39(0)2 8224 1

**Supplemental Table 1: Drugs categories, names and cohorts**

| Drug Category | Drug Name | Cohort | Drug Category | Drug Name | Cohort |
| --- | --- | --- | --- | --- | --- |
| Gastrointestinal agents | Metoclopramide | IV_Others drugs | Cardiovascular Drug | Urapidil | IV_Others drugs |
| Gastrointestinal agents | Pantoprazole | IV_Others drugs | Renal agents | Torasemide | IV_Others drugs |
| Gastrointestinal agents | Omeprazole | IV_Others drugs | Renal agents | Spironolactone | IV_Others drugs |
| Gastrointestinal agents | Debridat | IV_Others drugs | Renal agents | Etacrinic acid | IV_Others drugs |
| Gastrointestinal agents | Ondansetron | IV_Others drugs | Renal agents | Furosemide/Spironolactone | IV_Others drugs |
| Gastrointestinal agents | Ursodeoxycholic acid | IV_Others drugs | Renal agents | Lasix | IV_Others drugs |
| Gastrointestinal agents | Magnesium | IV_Others drugs | Renal agents | Canrenone | IV_Others drugs |
| Amnesic (anesthesia) | Diazepam | IV_sedative / analgesic | Cardiovascular Drug | Captopril | IV_Others drugs |
| Amnesic (anesthesia) | Lorazepam | IV_sedative / analgesic | Cardiovascular Drug | Carvedilol | IV_Others drugs |
| Amnesic (anesthesia) | Midazolam | IV_sedative / analgesic | Cardiovascular Drug | Clopidogrel | IV_Others drugs |
| Amnesic (anesthesia) | Alprazolam | IV_sedative / analgesic | Cardiovascular Drug | Digoxin | IV_Others drugs |
| Amnesic (anesthesia) | Clonazepam | IV_sedative / analgesic | Cardiovascular Drug | Diltiazem | IV_Others drugs |
| Analgesic (anesthesia) | Fentanyl | IV_sedative / analgesic | Cardiovascular Drug | Dobutamine | IV_Vasoactive drugs |
| Analgesic (anesthesia) | Hydromorphone | IV_sedative / analgesic | Cardiovascular Drug | Dopamine | IV_Vasoactive drugs |
| Analgesic (anesthesia) | Meperidine | IV_sedative / analgesic | Cardiovascular Drug | Enoximone | IV_Others drugs |
| Analgesic (anesthesia) | Morphin | IV_sedative / analgesic | Cardiovascular Drug | Heparin | IV_Others drugs |
| Analgesic (anesthesia) | Remifentanil | IV_sedative / analgesic | Cardiovascular Drug | Epinephrine | IV_Others drugs |
| Analgesic (anesthesia) | Sufentanil | IV_sedative / analgesic | Cardiovascular Drug | Eptifibatide | IV_Others drugs |
| Analgesic (anesthesia) | Tapentadol | IV_sedative / analgesic | Cardiovascular Drug | Esmolol | IV_Vasoactive drugs |
| Analgesic (anesthesia) | Tramadol | IV_sedative / analgesic | Cardiovascular Drug | Phenylephrine | IV_Others drugs |
| Antagonists | Flumazenil | IV_Others drugs | Cardiovascular Drug | Fleicainide | IV_Others drugs |
| Antagonists | Naloxone | IV_Others drugs | Cardiovascular Drug | Furosemide | IV_Others drugs |
| Antagnosists | Neostigmine/Instrastigmine | IV_Others drugs | Cardiovascular Drug | Isoprenaline | IV_Others drugs |
| Antagonists | Sugammadex | IV_Others drugs | Cardiovascular Drug | Labetalol | IV_Vasoactive drugs |
| Antagonists | Acetylcysteine | IV_Others drugs | Cardiovascular Drug | Lidocaine | IV_Others drugs |
| Antagonists | Oxicodone / Naloxone | IV_Others drugs | Cardiovascular Drug | Lisinopril | IV_Others drugs |
| anti-infective drugs | Acyclovir | IV_Antibiotics | Cardiovascular Drug | Metoprolol | IV_Others drugs |
| anti-infective drugs | Amphotericin B | IV_Antibiotics | Cardiovascular Drug | Milrinone | IV_Others drugs |
| anti-infective drugs | Amikacin | IV_Antibiotics | Cardiovascular Drug | Nistroglycerin | IV_Vasoactive drugs |
| anti-infective drugs | Amikacin | IV_Antibiotics | Cardiovascular Drug | Sodium nitropusside | IV_Vasoactive drugs |
| anti-infective drugs | Amoxicillin/Clavulanate | IV_Antibiotics | Cardiovascular Drug | Noradrenaline | IV_Vasoactive drugs |
| anti-infective drugs | Ampicillin | IV_Antibiotics | Cardiovascular Drug | Adrenaline | IV_Vasoactive drugs |
| anti-infective drugs | Ampicillin | IV_Antibiotics | Cardiovascular Drug | Norepinephrine | IV_Vasoactive drugs |
| anti-infective drugs | Azitromycin | IV_Antibiotics | Cardiovascular Drug | Procainamide | IV_Others drugs |
| anti-infective drugs | Aztreonam | IV_Antibiotics | Cardiovascular Drug | Streptokinase | IV_Others drugs |
| anti-infective drugs | Cefazolin | IV_Antibiotics | Cardiovascular Drug | Terlipressin | IV_Vasoactive drugs |
| anti-infective drugs | Cefazolin | IV_Antibiotics | Cardiovascular Drug | Vasopressin | IV_Vasoactive drugs |
| anti-infective drugs | Cefotaxime | IV_Antibiotics | Cardiovascular Drug | Verapamil | IV_Others drugs |
| anti-infective drugs | Ceftazidime+Avibactam (Zavicefta) | IV_Antibiotics | Cardiovascular Drug | Vitamin K | IV_Others drugs |
| anti-infective drugs | Ceftriaxone | IV_Antibiotics | Cardiovascular Drug | Warfarin | IV_Others drugs |
| anti-infective drugs | Ciprofloxacin | IV_Antibiotics | Hematological agents | Heparin | IV_Others drugs |
| anti-infective drugs | Clindamycin | IV_Antibiotics | Hematological agents | Protamine | IV_Others drugs |
| anti-infective drugs | Clindamycin | IV_Antibiotics | Immunosoppressants | Cyclosporin | IV_Others drugs |
| anti-infective drugs | Fluconazole | IV_Antibiotics | Immunosoppressants | Tacrolimus | IV_Others drugs |
| anti-infective drugs | Ganciclovir | IV_Antibiotics | Immunosoppressants | Methylprednisolone | IV_Others drugs |
| anti-infective drugs | Gentamicin | IV_Antibiotics | Hypnotics (anesthesia) | Ketamine | IV_sedative / analgesic |
| anti-infective drugs | Imipenem+Cilastatin | IV_Antibiotics | Hypnotics (anesthesia) | Propofol | IV_sedative / analgesic |
| anti-infective drugs | Levofloxacin | IV_Antibiotics | Hypnotics (anesthesia) | Sodium pentothal | IV_sedative / analgesic |
| anti-infective drugs | Meropenem | IV_Antibiotics | Myorelaxants (anesthesia) | Atracurium | IV_Others drugs |
| anti-infective drugs | Metronidazole | IV_Antibiotics | Myorelaxants (anesthesia) | Cisatracurium | IV_Others drugs |
| anti-infective drugs | Nystatin | IV_Antibiotics | Myorelaxants (anesthesia) | Pancuronium | IV_Others drugs |
| anti-infective drugs | Oxacillin | IV_Antibiotics | Myorelaxants (anesthesia) | Rocuronium | IV_Others drugs |
| anti-infective drugs | Penicillin | IV_Antibiotics | Myorelaxants (anesthesia) | Succinylcholine | IV_Others drugs |
| anti-infective drugs | Penicillin | IV_Antibiotics | Myorelaxants (anesthesia) | Vecuronium | IV_Others drugs |
| anti-infective drugs | Piperacillin | IV_Antibiotics | Neurological Drugs | Alfentanil | IV_sedative / analgesic |
| anti-infective drugs | Piperacillin+Tazobactam | IV_Antibiotics | Neurological Drugs | Haloperidol | IV_Others drugs |
| anti-infective drugs | Sulfamethoxazole/Trimethoprim (Bactrim) | IV_Antibiotics | Neurological Drugs | Atropine | IV_Others drugs |
| anti-infective drugs | Sulfisoxazole | IV_Antibiotics | Neurological Drugs | Caffeine citrate | IV_Others drugs |
| anti-infective drugs | Ticarcillin | IV_Antibiotics | Neurological Drugs | Diazepam | IV_sedative / analgesic |
| anti-infective drugs | Tobramycin | IV_Antibiotics | Neurological Drugs | Edrophonium | IV_Others drugs |
| anti-infective drugs | Vancomycin | IV_Antibiotics | Neurological Drugs | Etomidate | IV_sedative / analgesic |
| anti-infective drugs | Vancomycin | IV_Antibiotics | Neurological Drugs | Phenytoin | IV_Others drugs |
| anti-infective drugs | Anidulafungin | IV_Antibiotics | Neurological Drugs | Fentanyl | IV_sedative / analgesic |
| anti-infective drugs | Linezolid | IV_Antibiotics | Neurological Drugs | Physostigmine | IV_Others drugs |
| anti-infective drugs | Ceftobiprole | IV_Antibiotics | Neurological Drugs | Fluxetine | IV_Others drugs |
| anti-infective drugs | Teicoplanin | IV_Antibiotics | Neurological Drugs | Glycopyrrolate | IV_Others drugs |
| anti-infective drugs | Daptomycin | IV_Antibiotics | Neurological Drugs | Ibuprofen | IV_Others drugs |
| anti-infective drugs | Capsofungin | IV_Antibiotics | Neurological Drugs | Hydromorphone | IV_Others drugs |
| anti-infective drugs | Tigecycline | IV_Antibiotics | Neurological Drugs | Isoram | IV_Others drugs |
| anti-infective drugs | Cefepime | IV_Antibiotics | Neurological Drugs | Lorazepam | IV_sedative / analgesic |
| anti-infective drugs | Voriconazole | IV_Antibiotics | Neurological Drugs | Meperidine | IV_Others drugs |
| anti-infective drugs | Ceftolozane / Tazobactam (Zerbaxa) | IV_Antibiotics | Neurological Drugs | Midazolam | IV_sedative / analgesic |
| anti-infective drugs | Fosfomycin | IV_Antibiotics | Neurological Drugs | Morphin | IV_sedative / analgesic |
| anti-infective drugs | Oseltamvir | IV_Antibiotics | Neurological Drugs | Naloxone | IV_Others drugs |
| anti-infective drugs | Colistin | IV_Antibiotics | Neurological Drugs | Neostigmine/Instrastigmine | IV_Others drugs |
| anti-inflammatory | Ibuprofen | IV_Others drugs | Neurological Drugs | Paracetamol | IV_sedative / analgesic |
| anti-inflammatory | Ketoprofen | IV_Others drugs | Neurological Drugs | Pyridostigmine | IV_Others drugs |
| anti-inflammatory | Diclofenac | IV_Others drugs | Neurological Drugs | Propofol | IV_sedative / analgesic |
| anti-inflammatory | Ketorolac | IV_Others drugs | Neurological Drugs | Quetiapine fumarate | IV_Others drugs |
| Beta-blockers | Carvedilol | IV_Vasoactive drugs | Neurological Drugs | Remifentanil | IV_sedative / analgesic |
| Beta-blockers | Labetalol | IV_Vasoactive drugs | Neurological Drugs | Rocuronium | IV_Others drugs |
| Beta-blockers | Metoprolol | IV_Vasoactive drugs | Neurological Drugs | Scopolamine | IV_Others drugs |
| Beta-blockers | Atenolol | IV_Vasoactive drugs | Neurological Drugs | Vasopressin | IV_Others drugs |
| Beta-blockers | Bisoprolol | IV_Vasoactive drugs | Neurological Drugs | Clonidine |  |
| Cardiovascular (anesthesia) | Amiodarone | IV_Vasoactive drugs | Neurological Drugs | Carbamazepine | IV_Others drugs |
| Cardiovascular (anesthesia) | Atropine | IV_Others drugs | Neurological Drugs | Pregabalin | IV_Others drugs |
| Cardiovascular (anesthesia) | Calcium chloride | IV_Others drugs | Neurological Drugs | Valproic acid | IV_Others drugs |
| Cardiovascular (anesthesia) | Ephedrine | IV_Vasoactive drugs | Neurological Drugs | Levetiracetam | IV_Others drugs |
| Cardiovascular (anesthesia) | Epinephrine | IV_Vasoactive drugs | Neurological Drugs | Lacosamide | IV_Others drugs |
| Cardiovascular (anesthesia) | Esmolol | IV_Vasoactive drugs | Hormones / Antidiabetics | Dexamethasone | IV_Others drugs |
| Cardiovascular (anesthesia) | Phenylephrine | IV_Vasoactive drugs | Hormones / Antidiabetics | Sumatriptan succinate | IV_Others drugs |
| Cardiovascular (anesthesia) | Metoprolol | IV_Vasoactive drugs | Hormones / Antidiabetics | Long acting insulin | IV_Others drugs |
| Cardiovascular (anesthesia) | Nistroglycerin | IV_Vasoactive drugs | Hormones / Antidiabetics | Short acting insulin | IV_Others drugs |
| Cardiovascular (anesthesia) | Sodium nitropusside | IV_Vasoactive drugs | Hormones / Antidiabetics | Regular insulin | IV_Others drugs |
| Cardiovascular (anesthesia) | Norepinephrine | IV_Vasoactive drugs | Hormones / Antidiabetics | Ocreotide | IV_Others drugs |
| Cardiovascular (anesthesia) | Procainamide | IV_Vasoactive drugs | Hormones / Antidiabetics | Levothyroxine | IV_Others drugs |
| Cardiovascular (anesthesia) | Verapamil | IV_Vasoactive drugs | Other agents | Prothrombin complex | IV_Others drugs |
| Cardiovascular Drug | Abciximab | IV_Others drugs | Other agents | Fibrinogen | IV_Others drugs |
| Cardiovascular Drug | Adenosine | IV_Others drugs | Other agents | Vitamin B1 | IV_Others drugs |
| Cardiovascular Drug | Alteplase | IV_Others drugs | Other agents | Allopurinol | IV_Others drugs |
| Cardiovascular Drug | Amiodarone | IV_Vasoactive drugs | Other agents | Cernevit | IV_Others drugs |
| Cardiovascular Drug | Amlodipine | IV_Others drugs | Other agents | Magnesium sulfate | IV_Others drugs |
| Cardiovascular Drug | Amrinone | IV_Others drugs | Other agents | Gelofusine | IV_Others drugs |
| Cardiovascular Drug | Aspirin | IV_Others drugs | Other agents | Vitamin C | IV_Others drugs |
| Cardiovascular Drug | Atorvastatin | IV_Others drugs | Other agents | Electrolyte solution | IV_Others drugs |
| Cardiovascular Drug | Atropine | IV_Others drugs | Other agents | Isopuramin | IV_Others drugs |
| Cardiovascular Drug | Bivalirudin | IV_Others drugs | IV_sedative / analgesic | Hydroxyzine | IV_Others drugs |
| Cardiovascular Drug | Cangrelor | IV_Others drugs | Other agents | Tranexamic acid | IV_Others drugs |
| Cardiovascular Drug | Levosimendan | IV_Vasoactive drugs | Other agents | Calcium gluconate | IV_Others drugs |
| Cardiovascular Drug | Etilefrine | IV_Vasoactive drugs | Other agents | Mannitol | IV_Others drugs |
| Cardiovascular Drug | Methylene blue | IV_Others drugs | IV_sedative / analgesic | Dexmedetomidine | IV_sedative / analgesic |
| Cardiovascular Drug | Argipressin | IV_Vasoactive drugs |  |  |  |

**Supplemental Table 2. Type of fluids during ICU stay (data are all expressed in mL)**

|  | DAY 1 | DAY 2 | DAY 3 | DAY 4 | OVERALL |
| --- | --- | --- | --- | --- | --- |
| Albumine |  |  |  |  |  |
| Survivors | 29 ± 66 | 33 ± 65 | 23 ± 52 | 18 ± 49 | 95 ± 146 |
| Non-survivors | 46 ± 92 | 74 ± 127 | 40 ± 78 | 45 ± 70 | 167 ± 244 |
| Overall | 34 ± 75 | 44 ± 88 | 28 ± 61 | 25 ± 56 | 117 ± 184 |
| p-value* | 0.079 | 0.022 | 0.023 | 0.002 | 0.005 |
| Normal Saline |  |  |  |  |  |
| Survivors | 29 ± 182 | 74 ± 305 | 74 ± 305 | 4 ± 26 | 109 ± 439 |
| Non-survivors | 86 ± 276 | 147 ± 345 | 147 ± 345 | 56 ± 265 | 276 ± 625 |
| Overall | 46 ± 215 | 94 ± 318 | 15 ± 107 | 17 ± 137 | 159 ± 507 |
| p-value* | 0.001 | 0.008 | 0.208 | 0.437 | 0.003 |
| Reidratante III Solution* |  |  |  |  |  |
| Survivors | 765 ± 744 | 1007 ± 913 | 439 ± 652 | 317 ± 537 | 2390 ± 1951 |
| Non-survivors | 932 ± 1040 | 772 ± 864 | 502 ± 735 | 333 ± 564 | 2261 ± 1915 |
| Overall | 815 ± 845 | 940 ± 903 | 456 ± 674 | 322 ± 543 | 2351 ± 1937 |
| p-value* | 0.594 | 0.054 | 0.870 | 0.625 | 0.717 |
| Ringer Solution* |  |  |  |  |  |
| Survivors | 362 ± 732 | 494 ± 860 | 218 ± 490 | 149 ± 392 | 1163 ± 1615 |
| Non-survivors | 412 ± 885 | 454 ± 756 | 311 ± 647 | 178 ± 431 | 1176 ± 2012 |
| Overall | 377 ± 779 | 483 ± 831 | 244 ± 539 | 157 ± 402 | 1167 ± 1739 |
| p-value* | 0.848 | 0.717 | 0.429 | 0.626 | 0.893 |
| Other Fluids IV* |  |  |  |  |  |
| Survivors | 45 ± 114 | 141 ± 321 | 171 ± 345 | 208 ± 395 | 501 ± 946 |
| Non-survivors | 78 ± 151 | 128 ± 211 | 207 ± 357 | 266 ± 424 | 481 ± 725 |
| Overall | 55 ± 127 | 138 ± 294 | 181 ± 348 | 223 ± 402 | 510 ± 912 |
| p-value* | 0.076 | 0.250 | 0.090 | 0.341 | 0.137 |
| Other Fluids OS/NGT |  |  |  |  |  |
| Survivors | 507 ± 498 | 542 ± 592 | 572 ± 672 | 398 ± 756 | 1844 ± 1598 |
| Non-survivors | 319 ± 377 | 452 ± 461 | 406 ± 503 | 270 ± 444 | 1176 ± 1223 |
| Overall | 451 ± 472 | 518 ± 560 | 526 ± 633 | 366 ± 691 | 1639 ± 1521 |
| p-value* | 0.002 | 0.658 | 0.292 | 0.881 | 0.002 |

IV, intravenous; NGT, nasograstric tube.

*= Reidratante III and Ringer Solutions (Acetate and Lactate) have been considered together as “Balanced Solutions”, see Methods sections for further details.

“other fluids” include any type of glucosate, bicarbonate and blood products

*p values refer to the comparison between survivors and non-survivors.

**Supplemental Table 3. Categories of infusions during ICU stay (data are all expressed in mL)**

|  | DAY 1 | DAY 2 | DAY 3 | DAY 4 | OVERALL |
| --- | --- | --- | --- | --- | --- |
| IV antibiotics |  |  |  |  |  |
| Survivors | 142 ± 171 | 350± 279 | 338 ± 244 | 378 ± 269 | 1141 ± 805 |
| Non-survivors | 216 ± 216 | 466 ± 339 | 474 ± 352 | 522 ± 365 | 1460 ± 1101 |
| Overall | 164 ± 188 | 384 ± 302 | 375 ± 284 | 417 ± 304 | 1237 ± 913 |
| p-value* | 0.011 | 0.011 | 0.022 | 0.014 | 0.099 |
| IV other drugs |  |  |  |  |  |
| Survivors | 65 ± 94 | 196 ± 141 | 231 ± 186 | 243 ± 197 | 695 ± 479 |
| Non-survivors | 74 ± 94 | 210 ± 159 | 211 ± 136 | 277 ± 294 | 669 ± 513 |
| Overall | 68 ± 94 | 200 ± 147 | 226 ± 174 | 252 ± 227 | 687 ± 488 |
| p-value* | 0.208 | 0.772 | 0.819 | 0.914 | 0.566 |
| IV vasoactive drugs |  |  |  |  |  |
| Survivors | 60 ± 77 | 154 ± 160 | 97 ± 116 | 68 ± 102 | 354 ± 342 |
| Non-survivors | 74 ± 81 | 225 ± 200 | 180 ± 178 | 140 ± 176 | 548 ± 470 |
| Overall | 64 ± 78 | 175 ± 175 | 121 ± 141 | 88 ± 131 | 413 ± 394 |
| p-value* | 0.318 | 0.013 | 0.002 | < 0.001 | 0.002 |
| IV sedative /analgesic |  |  |  |  |  |
| Survivors | 80 ± 94 | 227 ± 185 | 226 ± 178 | 250 ± 207 | 725 ± 563 |
| Non-survivors | 69 ± 80 | 194 ± 189 | 195 ± 164 | 251 ± 211 | 614 ± 498 |
| Overall | 77 ± 90 | 217 ± 187 | 217 ± 176 | 250 ± 207 | 691 ± 545 |
| p-value* | 0.752 | 0.173 | 0.271 | 0.998 | 0.190 |

IV, intravenous. *p values refer to the comparison between survivors and non-survivors.

Supplemental Figure 1


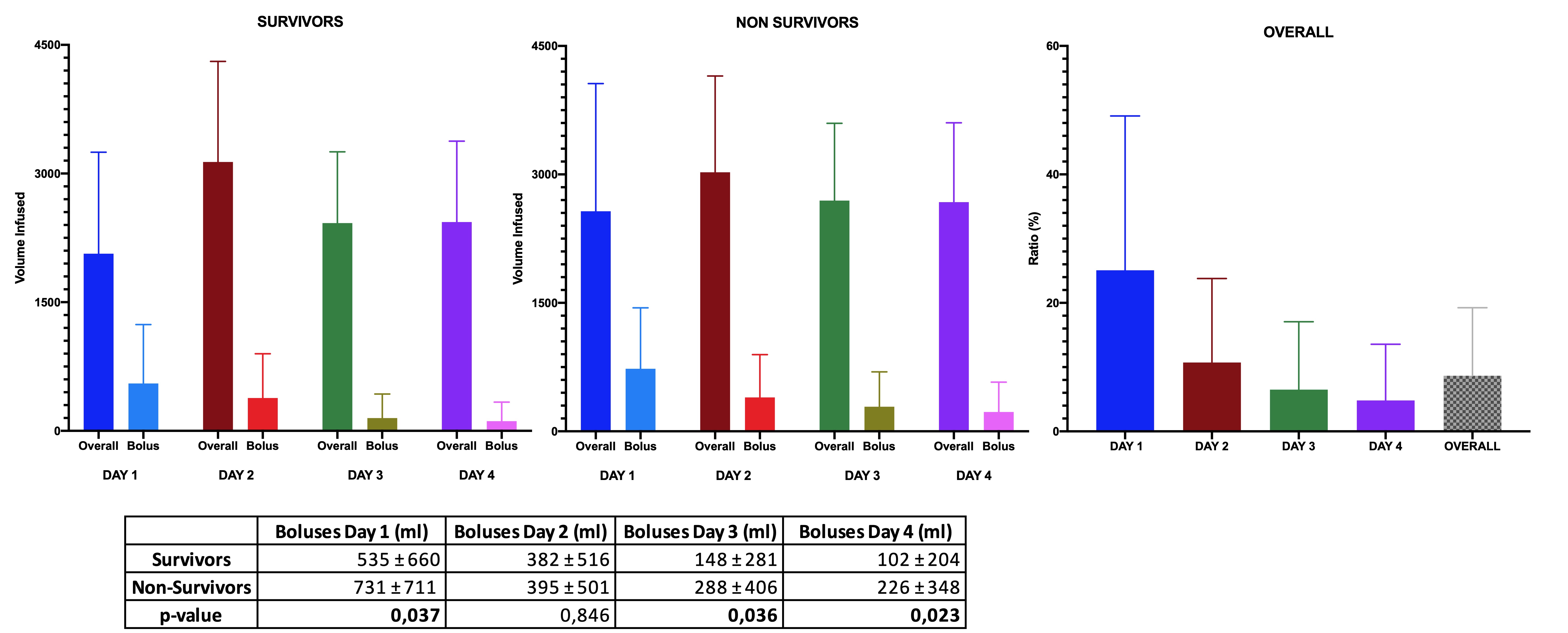


Volumes of fluid boluses in survivors and non-survivors (expressed as ml), and in the overall population (expressed as percentage between the amount of fluids given by boluses and the overall inputs of Days 1-4). Data are reported ad mean and standard deviation (SD). *p values refer to the comparison between survivors and non-survivors.
